# Development of V-Shaped Beam on the Shock Resistance and Driving Frequency of Micro Quartz Tuning Forks Resonant Gyroscope

**DOI:** 10.3390/mi11111012

**Published:** 2020-11-17

**Authors:** Bing Bai, Cun Li, Yulong Zhao

**Affiliations:** State Key Laboratory for Manufacturing Systems Engineering, Xi’an Jiaotong University, Xi’an 710049, China; bai402728119@stu.xjtu.edu.cn

**Keywords:** quartz tuning forks resonant gyroscope, shock response spectrum, half-cycle sine shock, modeling and simulation, shock test

## Abstract

The application of gyroscopes in harsh environments has always been a hot topic. As a high-quality material for manufacturing gyroscopes, quartz crystals need to be designed and optimized to meet the normal operation of gyroscopes in harsh environments. The Micro Electronics Mechanical System(MEMS) quartz tuning forks resonant gyroscope is one of the quartz gyroscopes. The elastic structure (V-shaped beam) between the anchor support point and tuning forks plays a vital role in the MEMS quartz tuning forks resonant gyroscope. This structure determines the natural frequency of the gyroscope, and more importantly, determines the shock resistance of the gyroscope structure. In this article, the MEMS quartz tuning forks gyroscope with different V-shaped beam thicknesses are simulated and analyzed by finite element analysis simulation software. After the modal analysis and shock simulation (the half-cycle sine shock pulse with amplitude of 1500 g (g is the acceleration of gravity) and duration of 2 ms in the six shock directions), the results show that when the beam thickness is 80 μm, the maximum stress is 94.721 MPa, which is less than the failure stress of quartz crystal. The gyroscope’s shock resistance is verified through shock testing.

## 1. Introduction

A gyroscope is a precision instrument that can sense the angular rate of rotatory objects. It is widely used in many fields, such as aerospace, navigation, automotive industry, and military [1,2,3]. Due to its many disadvantages, such as heavy weight, large size, and high price, traditional gyroscopes cannot be used in many specific occasions.

Recently, with the rapid growth of microelectronics technology and semiconductor process, Micro Electronics Mechanical System (MEMS) has gradually grown and emerged. At the same time, MEMS gyroscopes have also attracted the attention of researchers. The MEMS gyroscopes are cheaper [4], smaller, and lighter than traditional gyroscopes, and have comparable performance to traditional gyroscopes [5,6]. MEMS gyroscope devices are the world’s cutting-edge technology products, integrating micro-precision machinery, microelectronics, and semiconductor-integrated circuits. The MEMS quartz tuning forks resonant gyroscope is a kind of high-precision sensor in the MEMS Coriolis vibrating gyroscope.

At present, there are many companies in the world researching MEMS quartz tuning forks resonant gyroscopes and putting them into the market, including Microcomponents SA [7], Watson Industries [8,9], Toyota [10] BEI Systron Donner [11,12,13,14,15,16], etc. The MEMS quartz tuning forks resonant gyroscopes from these companies account for more than 90% of the military and civilian markets. With the continuous research and development of MEMS quartz tuning forks resonant gyroscopes, the application field of this gyroscope is becoming wider, and the application environment is also harsher and more complex. Sometimes, the gyroscope will operate in a very abominable environment, such as a high-shock environment. Shock is one of the most important factors affecting the reliability of MEMS. Unlike mechanical vibration stress, its acceleration amplitude is very high, the shock pulse width is small, and the shock waveform is like a half-sine wave or square wave pulse. In the actual application environment, the device may withstand great mechanical impact for example, when the device falls from a height of 1.5 m onto concrete ground, the shock acceleration is about 500~3500 g. The School of Electrical Engineering of Aalto University in Finland has conducted a shock test on a three-axis gyroscope. The failure modes of the mechanical structure after the shock mainly include comb tooth fracture, beam and structure fracture, and particle pollution [17,18,19]. To better analyze the shock resistance of the gyroscope and guide the design of the support and protection structure, it is necessary to model and analyze the impact response characteristics of the gyroscope. The Massachusetts Institute of Technology University and Beijing Institute of Technology University have pointed out that natural frequency, shock pulse width, acceleration amplitude, etc. have a greater impact on the shock reliability of MEMS devices [20,21]. The Binghamton University, State University of New York has established two-dimensional shock response models of Printed Circuit Board (PCB) and MEMS structures [22]. As producers of MEMS quartz tuning forks resonant gyroscopes, two companies have released the impact resistances of their different types of gyroscopes. BEI Systron Donner and Watson Industries have related reports [11,12,13,14,15,16]. While, Ref. [11] reported that the shock resistance of QRS11 micro-quartz gyroscope is 200 g, with a 10 ms half-sine shock pulse. Moreover, Ref. [12] reported that the company’s QRS28 micro-quartz gyroscope has a shock resistance of 300 g, with a 5 ms half-sine shock pulse. While, Ref. [13] found that the shock resistance of QRS2000 gyroscope to be 400 g, with a 2 ms half-sine shock pulse. Then, Refs. [14,15] reported that the shock resistance of QRS14 and SDG1400 gyroscope are both 200 g, with a 2 ms half-sine shock pulse. The authors in [16] reported that the shock resistance of QRS116 gyroscope is 1000 g, with a 2 ms half-sine shock pulse. The authors in [8,9] discussed the micro-quartz gyroscopes researched by Watson Industries. The shock resistance of ADS–C132, ADS–C232 and ARS–G152 micro-quartz gyroscopes is 500 g, with 10 ms half-sine shock pulse.

In this paper, a gyroscope and the structure connecting the tuning forks and the anchor point is introduced and analyzed. In addition, the measuring range is related to the shock resistance. The larger the range, the stronger the shock resistance. For this gyroscope, the range of the gyroscope is 300°/s. This gyroscope is used in fields such as automobiles and navigations. The V-shaped beam is a vital structure in the MEMS quartz tuning forks resonant gyroscope, it connects the elastic structures (drive tuning forks and detect tuning forks) and anchor support point and determines whether the gyroscope can work normally in a shock environment. The effect of the thickness of V-shaped beam on the gyroscope stability, natural frequency and shock resistance is simulated and analyzed in this paper. The thickness is determined by finite element simulation and verified by shock test after the simulation.

## 2. Working Principle and Fabrication Process of the Gyroscope

### 2.1. The Working Principle of the Gyroscope

**Figure 1:**
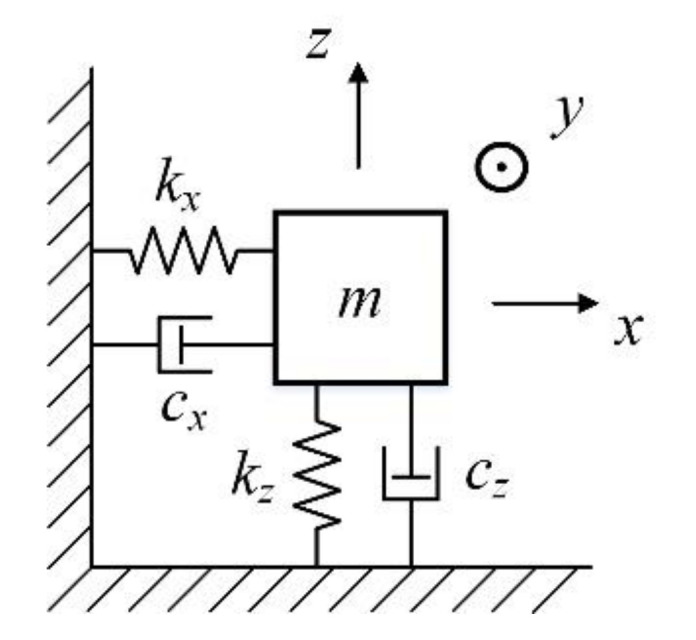
The model of ideal gyroscope.

In the ideal gyroscope model, the drive axis and the detection axis of the miniature quartz tuning fork gyroscope are orthogonal to each other. The mass-spring-damping system model of the ideal-gyroscope is simplified as shown in Figure 1. In the driving mode, the mass *m* is vibrated in the driving direction (*x*-axis). When the system is subjected to angular velocity, due to the Coriolis force, the mass *m* is vibrated in the detection direction (*z*-axis).

**Figure 2:**
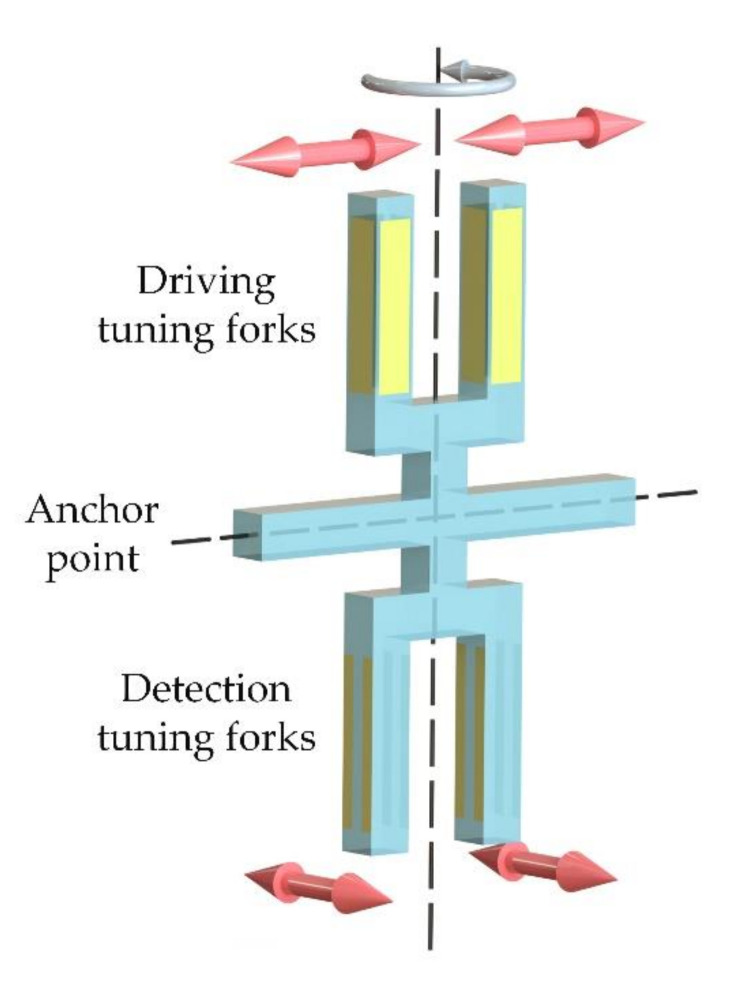
The principle of the tuning forks gyroscope.

**Figure 3:**
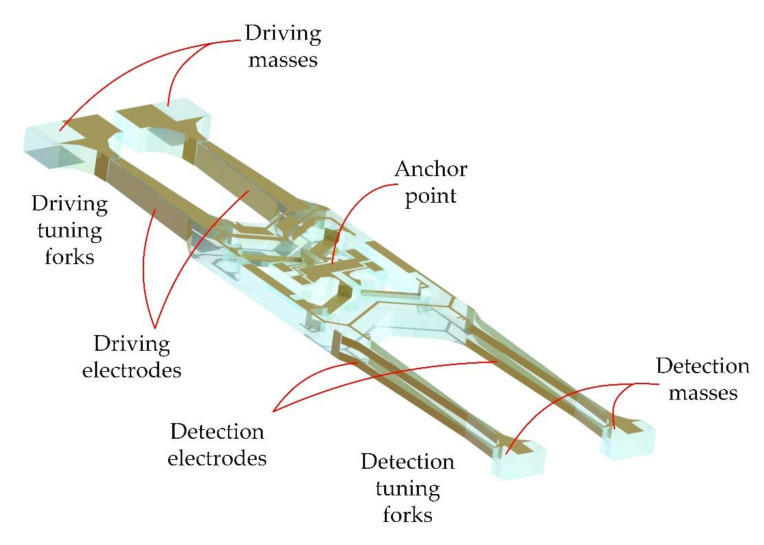
The structure of micro quartz tuning forks gyroscope.

The micro quartz tuning forks gyroscope is composed of two tuning forks, the driving tuning fork and the detecting tuning fork. Each tuning fork can be regarded as composed of two masses, as shown in Figure 2. Using the inverse piezoelectric effect of quartz crystals, the two masses in the driving tuning forks are vibrated toward each other (*x*-axis) near the resonance frequency of the driving mode by electrical signals. When the gyroscope rotates around the *y*-axis at an angular velocity Ω, the driving tuning fork is subjected to Coriolis force and generates vibration in a direction perpendicular to the plane of the tuning forks (*z*-axis). This Coriolis force movement is transmitted to the detection tuning fork by the supporting beams (V-shaped beams), which makes the detection tuning fork vibrate perpendicular to the plane of the tuning forks. The vibration signal of the detection tuning fork is converted into an electrical signal through the piezoelectric effect, and the electrical signal is proportional to the input angular velocity Ω. The electrical signal can calculate the input angular velocity through the signal conditioning circuit. For the micro quartz tuning forks gyroscope, the gyroscope structure of each part is shown in Figure 3.

### 2.2. Fabrication Process

**Figure 4:**
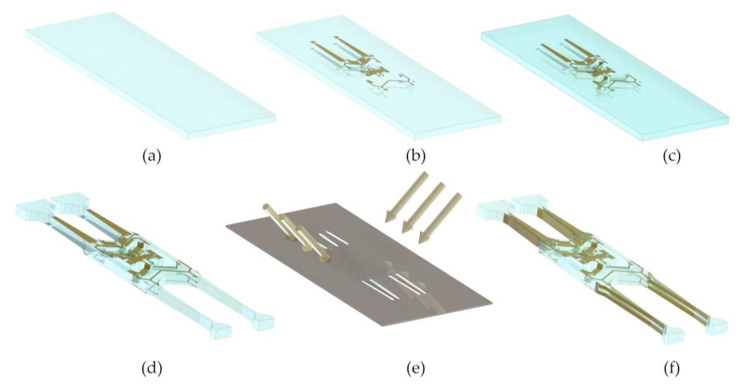
The fabrication process of micro quartz tuning forks gyroscope. (**a**) Z-cut quartz crystal wafers are prepared for organic cleaning and inorganic cleaning to ensure wafer cleanliness. (**b**) The photoresist is spin-coated on the front and back of the wafer, and photolithography is performed to form the pattern of the front electrode and the back electrode. Then, Cr and Au film are formed on the surface by magnetron sputtering, and the remaining metal is stripped. (**c**) Spin-coating photoresist and photolithography are performed again on the front and back of the wafer to form a window for etching the V-shaped beam followed by etching by Hydrofluoric acid (HF). Then, Cr and Au films were sputtered on the surface of the V-shaped beam. (**d**) The photoresist is sprayed on the front surface of the wafer, and patterned lithography of the tuning forks structure is performed; then, the tuning forks structure is formed by HF etching. (**e**) Using mechanical masking and lateral magnetron sputtering, the side electrodes of the tuning forks are formed. (**f**) The gyroscope sensitive structure is fabricated.

Fabrication of the micro quartz tuning forks gyroscope draws on MEMS process technology. The process mainly consists of the following steps: Cleaning, photolithography, magnetron sputtering, peeling and etching. The detailed steps are shown in Figure 4.

## 3. Analysis of Impact Response Characteristics of Micro-machined Gyroscope

**Figure 5:**
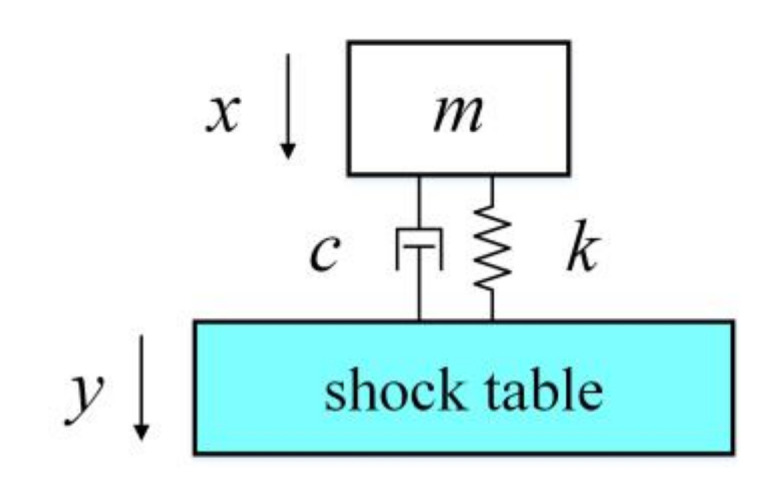
Single degree of freedom shock model.

Assuming that the quartz gyroscope structure is directly fixed on the shock table, as shown in Figure 5, and assuming that the installation structure of the gyroscope, the influence of PCB and adhesive glue are not considered and the impact dynamic equation of gyroscope in non-working state [23,24] is,

$$m\ddot{x}+c\left(\dot{x}-\dot{y}\right)+k\left(x-y\right)=0$$

where m, c, and k are the mass, damping coefficient, and stiffness of the quartz structure respectively.

Define $\omega =\sqrt{\frac{k}{m}}$ as the natural angular frequency of the gyroscope and $\xi =\frac{c}{2m\omega }$, $Q=\frac{1}{2\xi }$ are the damping ratio and quality factor *Q*, respectively. *x* and *y* are the absolute displacements of the gyroscope and the shock table, respectively, so the displacement *y* of the shock table is,

(1) $$y=y_{i}\sin\left(\omega_{i}t\right)$$

where $\omega_{i}$ is the excitation frequency of the shock table and $y_{i}$ is the displacement amplitude. After calculation, the response of the system can be calculated.

Define *z = x − y*, and *z*(*t*) can be obtained,

(2) $$z\left(t\right)=\left\{\begin{array}{cc} R\left(t\right) & 0<t\leq \tau \\ R\left(t\right)+R\left(t-\tau \right) & t>\tau \end{array}\right.$$

where

$$R\left(t\right)=\frac{\omega_{i}A}{\omega \sqrt{1-\xi^{2}}}e^{-\frac{\omega }{2Q}t}\sin\left(\sqrt{1-\xi^{2}}\omega t+\theta \right)+A\sin\left(\omega_{i}t-\varphi \right)$$

$$A=\frac{y_{i}\omega_{i}^{2}}{\omega^{2}\sqrt{{\left(1-\frac{\omega_{i}^{2}}{\omega^{2}}\right)}^{2}+{\left(\frac{\omega_{i}}{Q\omega }\right)}^{2}}}=\frac{y_{i}\omega_{i}^{2}}{\sqrt{\omega_{i}^{4}+2\left(2\xi^{2}-1\right)\omega^{2}\omega_{i}^{2}+\omega^{4}}}$$

φ=tg−1ωωiQω2−ωi2, and θ=tg−11−ξ2ξ−Qω2−ωi2ω2

From the above formulas, *R*(*t*) is related to *y_i_*, *ω_i_*, *ω*, and *ξ*. These parameters are very critical in the simulation process and need to be added in the simulation software.

## 4. Theory of Shock Test

Shock usually refers to a single interaction between the shock object and the research object, which can provoke a sudden change in the force, acceleration, velocity, or displacement of the transient disturbance and release a considerable amount of energy in a small space. The outstanding feature of the mechanical system’s response to shock excitation is that there is still a mechanical motion after the input action ends. The motion generated during the mechanical system and excitation is called the initial shock response; the motion that still exists after the end of the excitation is called the residual shock response. The shock motion process is distinguished from the vibration process by its single-time, non-periodic, and non-zero amplitude characteristics.

**Figure 6:**
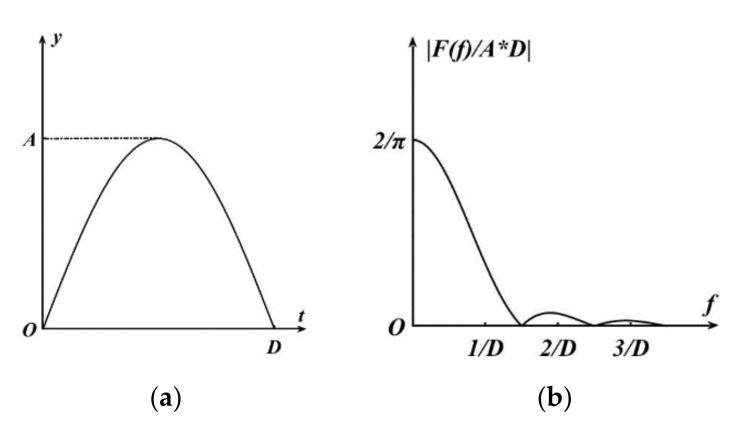
(**a**) Half-cycle sine shock pulse of amplitude *A* and pulse duration *D*. (**b**) Spectrum of the half-cycle sine shock pulse.

Generally, force, displacement, or acceleration are used to express the characteristics of shock. Among them, in most cases, acceleration is used to represent shock. The most common single pulse acceleration waveforms are half-cycle sine wave, rectangular wave, triangle wave, and clock wave. In this shock environment test, half-cycle sine shock pulse is used, and Figure 6a shows its pulse waveform. The math expression is,

(3) $$y(t)=\left\{\begin{array}{cc} A\cdot \sin\frac{\pi }{D}t & \left(0\leq t\leq D\right)\\ 0 & \left(t<0,t>D\right) \end{array}\right.$$

where *A* is the amplitude of the half-cycle sine shock pulse, and *D* is the pulse duration.

In the actual situation, when the shock pulse acts on the gyroscope, a waveform like a half-cycle sine wave pulse emerges at the beginning. However, these do not immediately vanish, and a series of instantaneous oscillation waveforms emerge before the waveform gradually disappears. This process is difficult to express with a clear mathematical expression, and it is usually necessary to convert the expression of the shock motion in the time domain into the frequency domain-that is, Fourier transform of the shock function. Its Fourier transform is:

(4) $$F(\omega )={\int_{-\infty }^{\infty }y(t)e}^{-j\omega t}dt.$$

when the shock is a half-cycle sine pulse, its spectrogram is shown in Figure 6b. Following Fourier transform, the obtained spectrum function is:

(5) $$\left|F(f)\right|=\frac{2AD}{\pi }\left|\frac{\cos\pi fD}{1-4f^{2}D^{2}}\right|.$$

Shock response spectrum is a concept commonly used in engineering [25]. The International Electrotechnical Commission (IEC), the International Organization for Standardization (ISO), and China’s national standards have all adopted the shock spectrum as one of the methods for specifying the shock environment. Therefore, the shock response spectrum is the basis for analyzing the shock resistance design of the equipment and the basic parameter of the simulation experiment for controlling the shock environment of the device. The device is subjected to shock, and the response of its shock response means that the product has the maximum stress-that is, the test sample has the largest deformation. Therefore, the maximum acceleration *a_max_* of the shock response is directly related to the damage and failure caused by the shock of the device, which leads to the maximum shock response spectrum.

**Figure 7:**
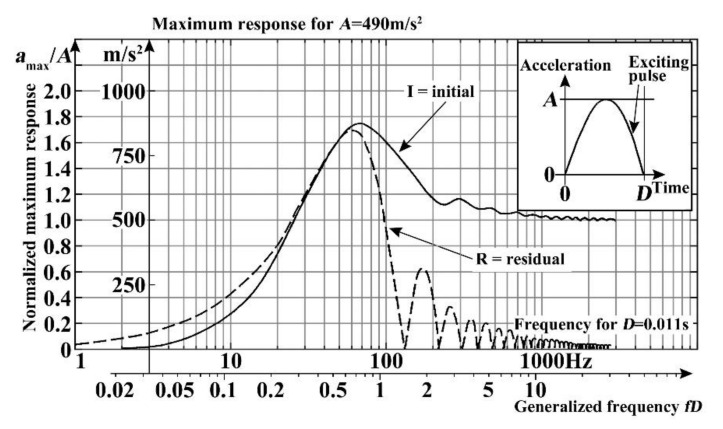
Shock response spectrum of half-cycle sine shock pulse (amplitude is 50 g and duration is 11 ms).

There are two ways to obtain the shock response spectrum, which are measured through experiments, and calculated by the known half-cycle sine response spectrum. Figure 7 shows the half-cycle sine shock pulse (the amplitude *A* is 50 g, and the duration *D* is 11 ms) shock response spectrum of an undamped single degree of freedom linear system given in the International Electrotechnical Commission (IEC) international standard shock test method.

The generalized frequency axis and the normalized maximum response axis are located below the frequency axis, and left of the maximum response axis, respectively. The shock response spectrum of normalized coordinate can be used to convert the half-cycle sine shock response spectrum of different *A* and *D* values. The converted expression is,

(6) $$\begin{array}{l} a(a)=\frac{a_{\max}}{A}\\ a=f_{n}\cdot D \end{array}$$

where *a*_max_ is acceleration maximum response, *A* is shock amplitude, *D* is shock duration, and *f_n_* is natural frequency of the system.

## 5. Modal Analysis of the Tuning Forks Structures

Vibration modes are inherent and integral characteristics of elastic structures. Through the modal analysis method, the characteristics of the main modes of the structure in a certain susceptible frequency range can be analyzed, and the actual vibration response of the structure under various external or internal vibration sources in this frequency band can be predicted. Therefore, modal analysis is an important method for structural dynamic design and equipment fault diagnosis.

**Figure 8:**
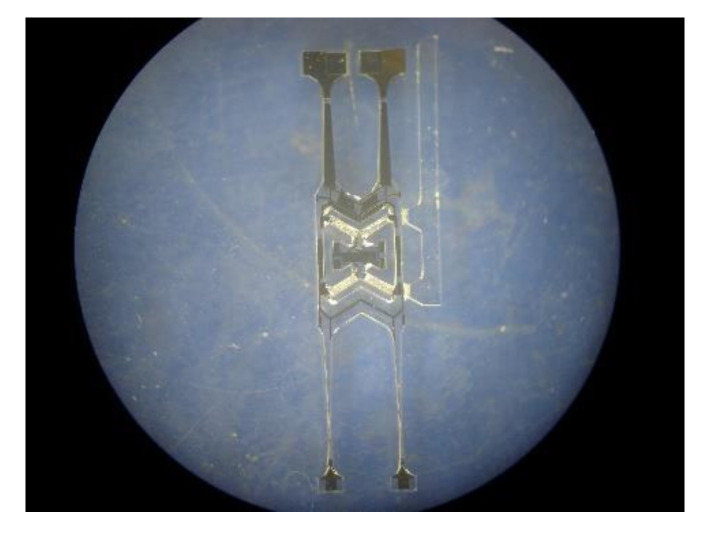
The sensitive unit of the Micro Electronics Mechanical System(MEMS)quartz resonant gyroscope.

**Figure 9:**
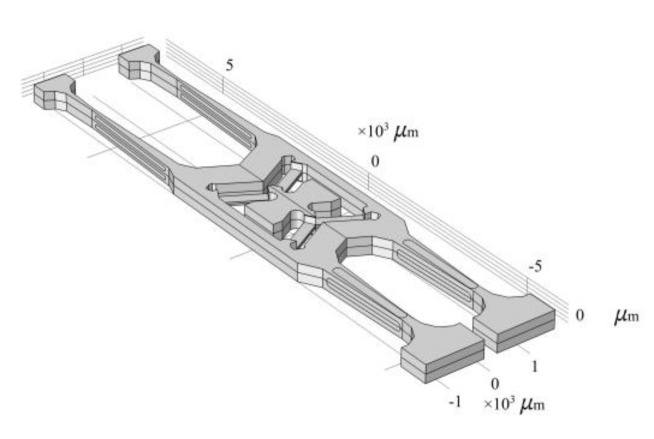
The quartz tuning forks resonant structure.

**Figure 10:**
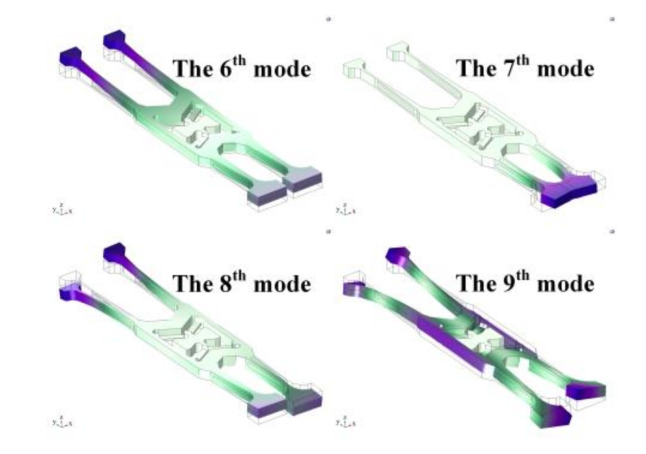
The 6th to 9th modal shapes of tuning forks resonant gyroscope.

**Figure 11:**
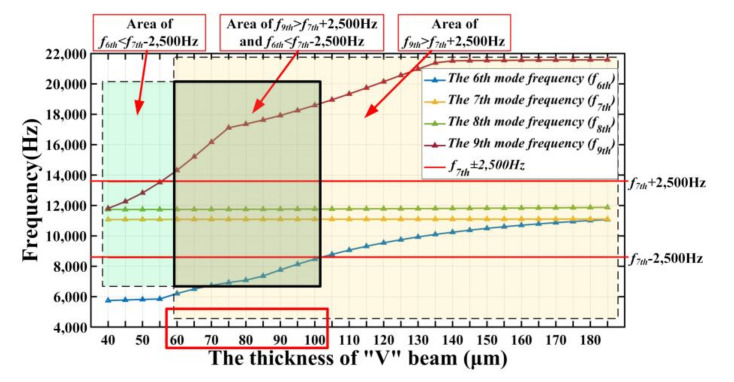
*f_6th_*, *f*_7*th*_, *f*_8*th*_, and *f*_9*th*_ of tuning forks resonant gyroscope models with different V-shaped beam thicknesses.

The sensitive unit of the quartz tuning forks resonant gyroscope fabricated by MEMS process is shown in Figure 8. The quartz tuning forks structure model is established, based on the actual structure in the COMSOL Multiphysics(COMSOL Inc, Burlington, MA, USA) finite element simulation software, as shown in Figure 9. The length of the gyroscope is about 12,000 μm, the width is about 3000 μm, and the thickness is about 400 μm. Then, modal analysis is performed on the model. Through simulation, the sixth to ninth modal shapes and corresponding resonance frequencies of tuning forks resonant gyroscope models with varying beam thicknesses are obtained, as shown in Figure 10 and Figure 11. Among them, the seventh mode and the eighth mode correspond to the working mode and detection mode of the gyroscope, respectively.

As there is random vibration in the frequency range of 20~2000 Hz in the working environment of the gyroscope, the frequency of the random vibration (*f_r_*) will be superimposed on the driving frequency (the seventh frequency, *f*_7*th*_) to generate another frequency. As *f_r_* is a random value between 20 Hz and 2000 Hz, the superimposed frequency (*f_r_ + f*_7*th*_) is also a random frequency within a frequency range. Significantly, if there are sixth resonance frequency (*f*_6*th*_) and ninth resonance frequency (*f*_9*th*_) within this range, it is possible to generate an output signal when the gyroscope has no angular velocity input. To ensure that the gyroscope is not affected by random vibration, *f*_6*th*_ and *f*_9*th*_ should not appear in this frequency range. To better ensure that the gyroscope works normally under random vibration, this frequency range is defined as (*f_7th_* − 2500) to (*f_7th_* + 2500). From Figure 11, the beam thickness range that meets the above requirements is from 60 μm to 100 μm.

Through modal analysis, it is determined that the initial V-shaped beam thickness range is from 60 μm to 100 μm. This is also the range of the thickness of the shock simulation beam in Section 6.

## 6. Modeling, Simulation and Analysis

### 6.1. Modeling of MEMS Quartz Tuning Forks Resonant Gyroscope

**Figure 12:**
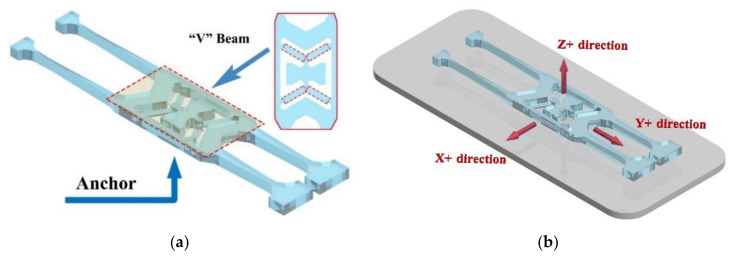
(**a**) Sensitive component model of the gyroscope. (**b**) Three of the six shock directions of shock simulation.

The V-shaped beam structure is extremely important structure to the shock resistance design of the gyroscope. It connects the anchor support point and the vibration tuning forks together, as shown in Figure 12a. In the second part, the initial thickness of the V-shaped beam has been determined to be 60 μm to 100 μm.

**Figure 13:**
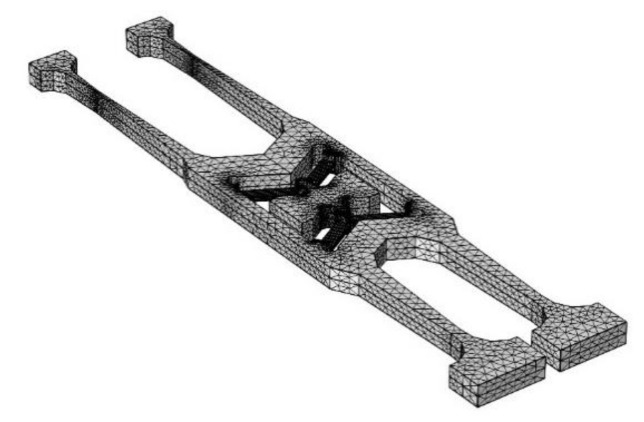
Meshing of the gyroscope model.

The gyroscope vibration structure model built by COMSOL Multiphysics is shown in Figure 12a. The quartz material used in the modeling is the same as the actual material, which is a Z−cut left-handed piezoelectric quartz crystal. The properties of the quartz are shown in Table 1 [26]. The quality factor *Q* of the gyroscope vibrating structure is about 17,607 under the test of the vacuum with the E4990A Impedance Analyzer. The relationship between *Q* and the damping ratio *ξ* is $Q=\frac{1}{2\xi }$, and we calculate the damping ratio *ξ* to be about 3 × 10^−5^. So, the damping ratio is 3 × 10^−5^ in the simulation software. In an actual situation, the gyroscope vibrating structure is fixed on the base by anchor points, so the anchor points of the model are set to be fixed constraints, and the remaining boundaries can be free. The entire gyroscope’s vibrating structure is supported by V-shaped beams. Next, the model is meshed. Since the parts of interest are the V-shaped beams, they need to be more densely meshed, as shown in Figure 13.

Before analyzing the shock resistance of this tuning fork gyroscope model, it is necessary to analyze the modal of this model and find the natural frequencies of the first four order modals. Then the shock pulse of the gyroscope model in six shock directions (X+ direction, X− direction, Y+ direction, Y− direction, Z+ direction, Z− direction) is simulated. Three of the six shock directions are shown in Figure 12b. The amplitude *A* of the half-cycle sine wave pulse is equal to 1500 g, and the pulse duration *D* is equal to 0.002 s. According to Formula (3) in Section 4, the function of the shock is:

(7) $$y(t)=\left\{\begin{array}{cc} 1500g\cdot \sin\frac{\pi }{0.002}t & \left(0\leq t\leq 0.002s\right)\\ 0 & \left(t<0,t>0.002s\right) \end{array}\right..$$

According to the Formula (6), the *a*_max_ and *f_n_* of this shock response spectrum is:

(8) $$\left\{\begin{array}{c} a_{\max,1500g}=30\cdot a_{\max,50g}\\ f_{n,1500g}=55\cdot f_{n,50g} \end{array}\right..$$

**Figure 14:**
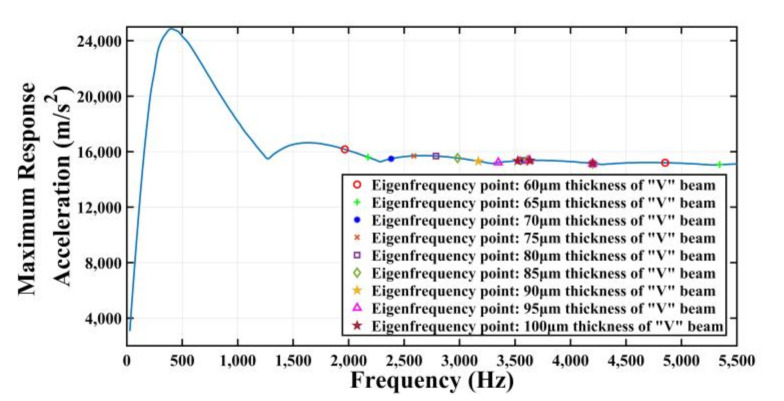
The shock response spectrum of half-cycle sine shock pulse (amplitude is 1500 g and duration is 2 ms) and the frequency points in Table 2.

Input this shock response spectrum is inputted into COMSOL Multiphysics, then the spectrum is drawn as shown in Figure 14.

### 6.2. Simulation and Analysis

This part introduces transient dynamics simulation and shock response spectrum simulation. Transient dynamic analysis is to determine the displacement, stress, strain and force of the structure under the random combination of static load, transient load and simple harmonic load. Shock spectrum analysis provides an analysis function that is different from transient response. In the analysis, the excitation of the structure is represented by small components, and the response of the structure to these components is the combination of the maximum response of each mode of the structure.

#### 6.2.1. Transient Dynamics Simulation

**Figure 15:**
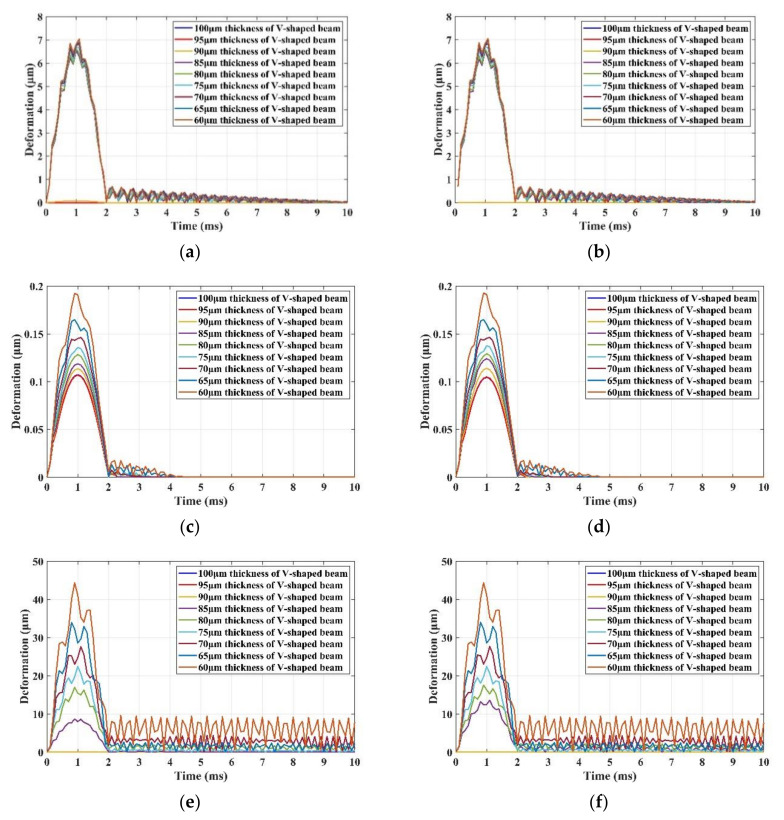
Model of deformation in six shock directions in transient dynamics simulation. (**a**,**b**) are the deformation in X+ and X– direction, respectively. (**c**,**d**) are the deformation in Y+ and Y− direction, respectively. (**e**,**f**) are the deformation in Z+ and Z− direction, respectively.

In transient dynamics simulation, the time range of transient simulation is 0~10 ms, the shock is 2 ms and the half-sine shock pulses in six shock directions, the shock pulse starts from 0 ms and lasts for 2 ms. When the shock is loaded on the model within the first 2 ms, the deformation generated by the model increases, and the deformation is the largest at around 1 ms. After the shock duration has passed, the model still vibrates and the deformation becomes smaller and smaller, as shown in Figure 15. Figure 15e,f show that when the shock direction is in the Z+ direction and Z− direction, the deformation produced by the model is much larger than the deformation produced by several other directions. Therefore, the impact resistance in the Z direction determines the shock resistance of the model.

#### 6.2.2. Shock Spectrum Analysis

**Figure 16:**
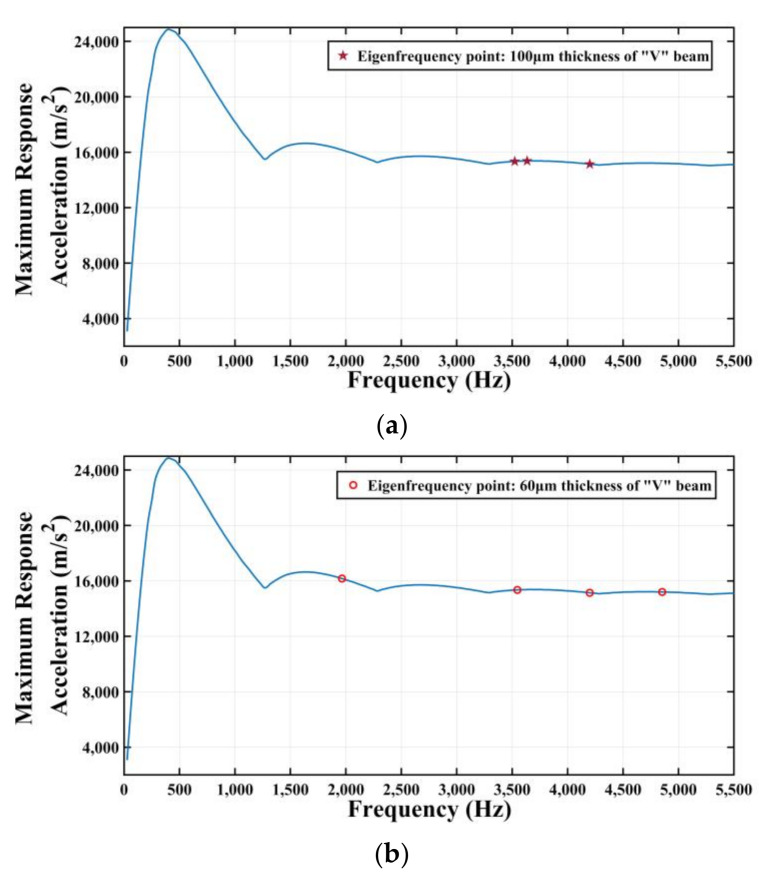
(**a**) Natural frequency distribution with 100 μm thickness of V-shaped beam. (**b**) Natural frequency distribution with 60 μm thickness of V-shaped beam.

Figure 14 shows that the frequency range of the given shock response spectrum is from 0 to 5500 Hz. Further, through the modal analysis of the gyroscope, the first fourth natural frequency of many kinds of V-shaped beam thicknesses are obtained, as shown in Table 2. All the natural frequency values within from 0 to 5500 Hz in Table 1 are placed in Figure 14. The natural frequency values of the MEMS tuning forks resonant gyroscope models with beam thicknesses of 100 μm and 60 μm are distributed in this shock response spectrum as shown in Figure 16a,b. As can be seen from Figure 16a, when the beam thickness is 100 μm, there are frequency points of three modes in the range of 0 to 5500 Hz; from Figure 16b, when the beam thickness is 60 μm, there are frequency points of four modes in the range of 0 Hz to 5500 Hz.

The shock response spectrum of 2 ms and six shock directions half-cycle sine shock pulse are applied to the structure with the V-shaped beam of many kinds of thicknesses (60 μm, 65 μm, 70 μm, 75 μm, 80 μm, 85 μm, 90 μm, 95 μm, 100 μm). Through the simulation analysis, the stress distribution and maximum stress value of these gyroscope modals during the half-cycle sine shock pulse are obtained.

**Figure 17:**
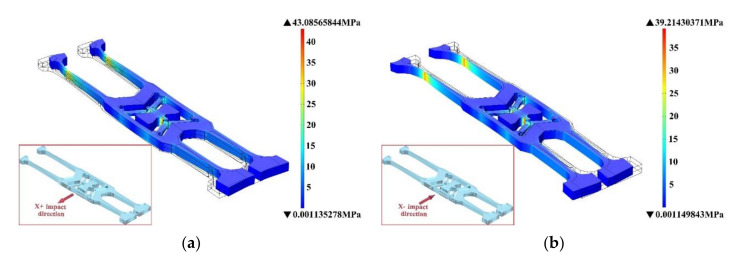

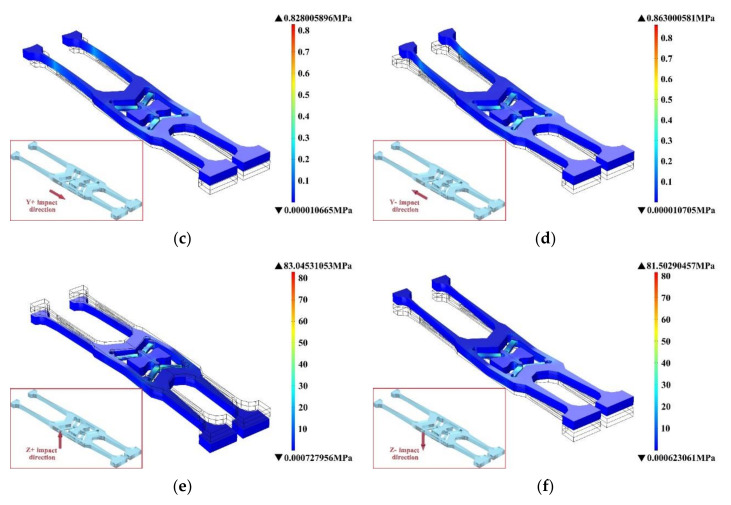
The stress contour with V-shaped beam thickness of 100 μm after exerting (**a**) X+ shock direction, (**b**) X− shock direction, (**c**) Y+ shock direction, (**d**) Y− shock direction, (**e**) Z+ shock direction, and (**f**) Z− shock direction.

When the V-shaped beam thickness is 100 μm and the structure is exerted on the shock response spectrums of six shock directions, the structural deformation and stress distribution are shown in Figure 17. Figure 17a,b indicate that when the X direction (X+ direction or X− direction) shock is applied on this structure, the stress at the thinner part of the detection of the tuning forks and the connection between the V-shaped beam and the anchor point are larger. Figure 17c,d show that when the Y direction (Y+ direction or Y− direction) shock is applied, the stress is smaller than the stress generated by the shock in the other two shock directions. Figure 17e,f show that when the shock in the Z direction (Z+ direction or Z− direction) is applied, the stress at the connection between the V-shaped beam and the anchor point is the largest compared to the stress generated by the shock in the X and Y directions—and it is more likely to break.

**Figure 18:**
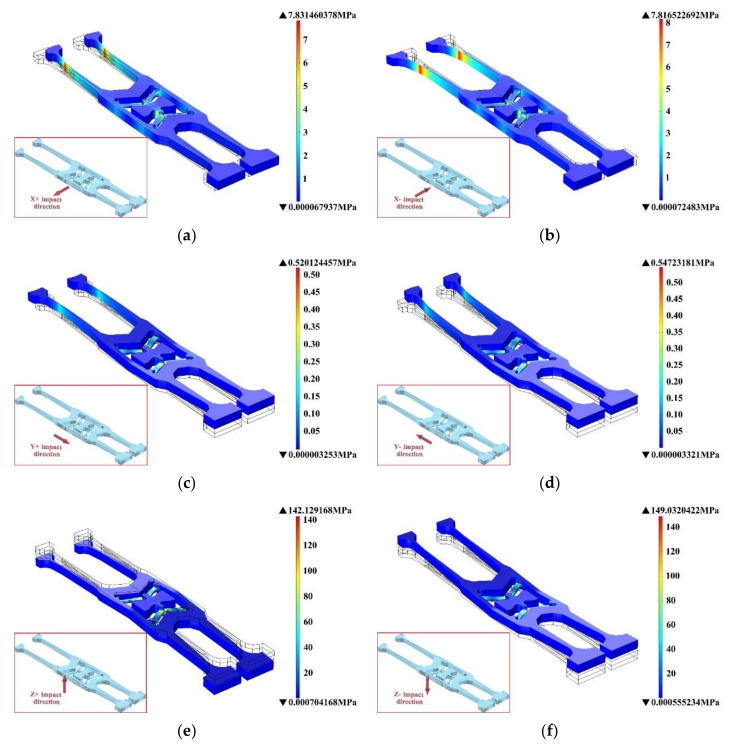
The stress contour with V-shaped beam thickness of 60 μm after exerted (**a**) X+ shock direction, (**b**) X− shock direction, (**c**) Y+ shock direction, (**d**) Y− shock direction, (**e**) Z+ shock direction and (**f**) Z− shock direction.

When the V-shaped beam thickness is 60 μm, the structural deformation and stress distribution are shown in Figure 18. From the overall point of view, when the V-shaped beam thickness is 60 μm, the stress generated after the specified shock is significantly larger than the stress generated at 100 μm. Therefore, the thinner the V-shaped beam, the larger the stress generated during shock, and the more likely the structure will be destroyed.

The maximum stress value with different V-shaped beam thicknesses are shown in Table 3. When the gyroscope is shocked in six shock directions, the maximum stress values for all V-shaped beam thicknesses are shown in Table 3. When the V-shaped beam thickness is 100 μm, the maximum stress value is 83.045 MPa, and it emerges in the Z+ shock direction. When the V-shaped beam thickness is 60 μm, the maximum stress value is 149.32 MPa, and it emerges in the Z− shock direction. When the V-shaped beam thickness is 80 μm, the stress value emerged by the shock in the Z− shock direction is the largest at 94.721 MPa; when the V-shaped beam thickness is 75 μm, the stress value emerged by the shock in the same direction is the largest, at 100.512 MPa. Limited by the fracture strength of quartz crystal materials (95 MPa) [27,28], the minimum thickness of the V-shaped beam is 80 μm.

## 7. Test and Experiment

Before undergoing the high-acceleration shock test, the driving frequency (*f_7th_*) of the MEMS quartz tuning forks resonant gyroscope with a V-shaped beam thickness of 80 μm analyzed and tested by the E4990A Impedance Analyzer is 11.104 kHz. The driving frequency (*f_7th_*) obtained by the simulation is 11.095 kHz.

**Figure 19:**
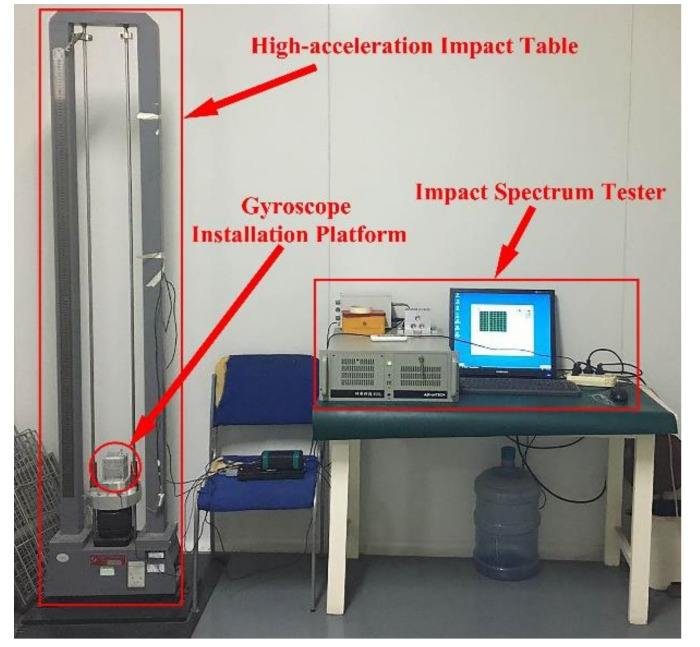
High-acceleration shock test table.

**Figure 20:**
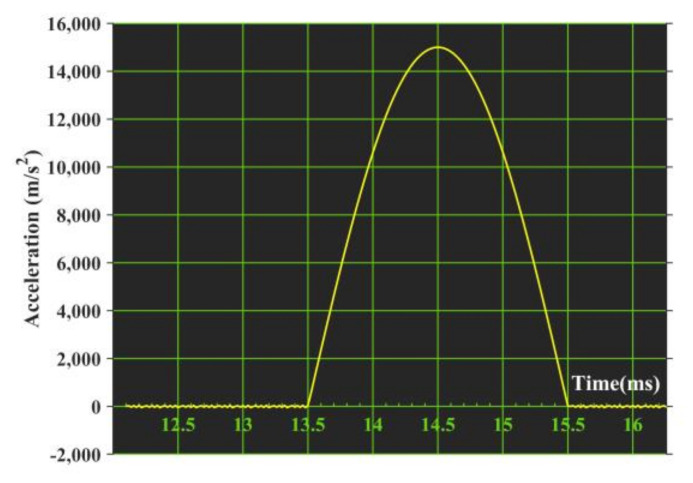
Half–sine wave.

At room temperature, the gyroscopes with V-shaped beam thicknesses of 80 μm and 75 μm are installed on the high-acceleration table according to the requirements, as shown in Figure 19; the gyroscopes are turned on; and the shock conditions of half-sine wave with an amplitude of 1500 g and duration time of 2 ms are exerted on the gyroscopes in six shock directions after the gyroscope has reached normal operating states. The shock pulse is shown in Figure 20.

A total of 10 gyroscopes with V-shaped beam thickness of 80 μm and 10 gyroscopes with V-shaped beam thickness of 75 μm are tested by the high-acceleration shock test, and the results are the same. The test results of two gyroscopes are explained in this section.

**Figure 21:**
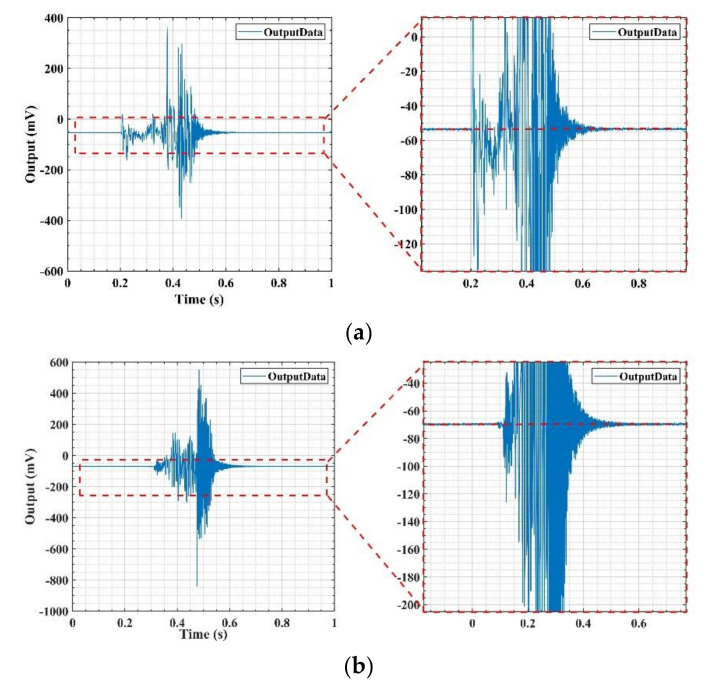
Output of gyroscope with V-shaped beam thickness of 80 μm after (**a**) Z+ shock direction and (**b**) Z− shock direction.

**Figure 22:**
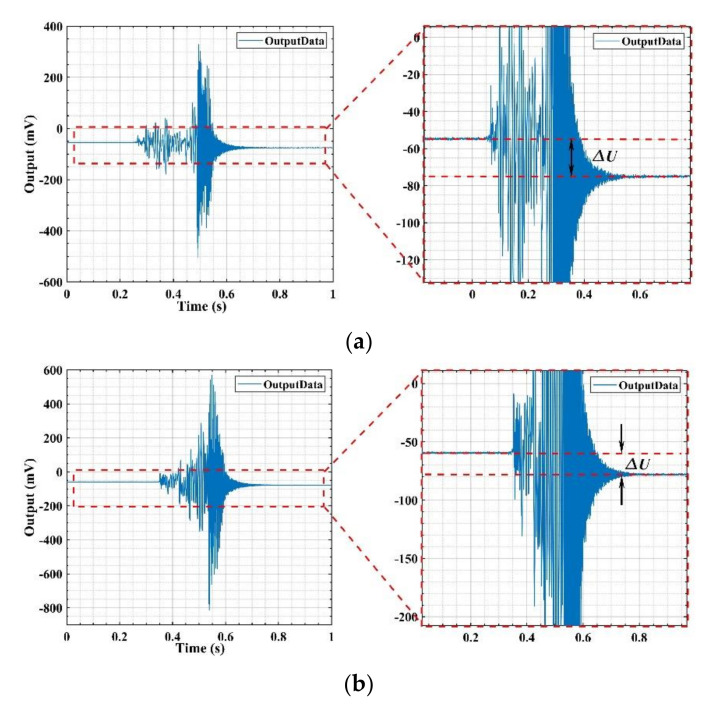
Output of gyroscope with V-shaped beam thickness of 75 μm after (**a**) Z+ shock direction and (**b**) Z− shock direction.

**Figure 23:**
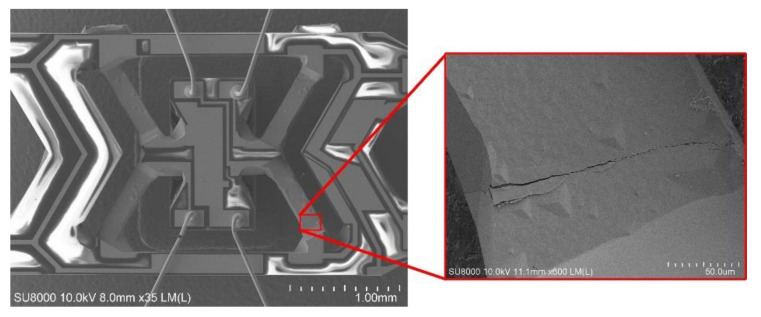
The broken V-shaped beam after the shock.

The test results found that when the shock in the Z+ or Z− directions are exerted, the output results of the gyroscopes with two V-shaped beam thicknesses are different. Figure 21a,b show the output of the gyroscope with a V-shaped beam thickness of 80 μm in the whole Z+ and Z− shock direction process, respectively. The results indicate that the output after the shock is equal to the output before the shock. The structure of the gyroscope is not destroyed when it is shocked. Figure 22a,b show the output of the gyroscope with a V-shaped beam thickness of 75 μm in the whole Z+ and Z− shock direction process. The results indicate that the output after shocking is not equal to the output before the shock and has a deviation, *ΔU*, as shown in Figure 22. The structure of gyroscope with a V-shaped beam thickness of 75 μm is destroyed by the shock from the experiment results. Further, as shown in Figure 23, the V-shaped beam with thickness of 75 μm is broken after the shock.

## 8. Conclusions

The simulation of shock resistance is carried out in six directions, and the shock resistance experiment is tested in the same six directions. The stress distributions and deformations exerted by the half-cycle sine shock pulse (*A* is 1500 g and *D* is 2 ms) in six shock directions are obtained by simulation and analysis of many kind of V-shaped beam thicknesses of the MEMS quartz tuning forks resonant gyroscope. Simulation results show that when the V-shaped beam thickness is 80 μm, the driving frequency is 11.104 kHz, and the maximum stress value emerged by the shock is 94.721 MPa. It is verified that the gyroscope with a V-shaped beam thickness of 75 μm is failed after the shock by experiments. The experimental results are consistent with the simulation results. The existing shock resistance technology is compared in Table 4.

## Figures and Tables

**Table 1:** Quartz crystal parameters.

Quantity	Symbol	Value
Density	*ρ*	2651 (kg/m^3^)
Young’s modulus	*E*	78.3 (GPa)
Poisson ratio	*ν*	0.14
Compliance matrix	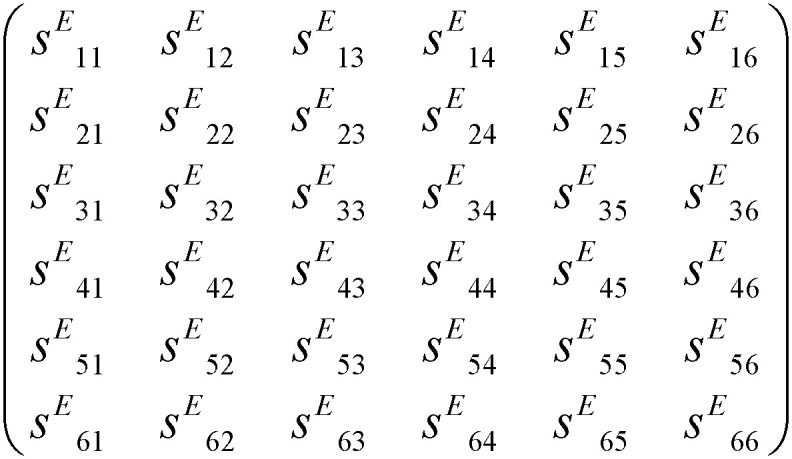	$\left(\begin{array}{cccccc} 12.77 & -1.790 & -1.220 & 4.5 & 0 & 0\\ -1.790 & 12.77 & -1.220 & -4.5 & 0 & 0\\ -1.220 & -1.220 & 9.6 & 0 & 0 & 0\\ 4.5 & -4.5 & 0 & 20 & 0 & 0\\ 0 & 0 & 0 & 0 & 20 & 9.0\\ 0 & 0 & 0 & 0 & 9.0 & 29.12 \end{array}\right)\times 10^{-12}(1/Pa)$

**Table 2:** The first 4th natural frequency of the structure with different V-shaped beam thicknesses.

Modal	The Natural Frequency of the Structure with Different V-Shaped Beam Thicknesses (Hz)								
	100 μm	95 μm	90 μm	85 μm	80 μm	75 μm	70 μm	65 μm	60 μm
1st	3523.2	3348.8	3169.1	2981.8	2787.7	2588.3	2383	2174.4	1965.1
2nd	3634.6	3626.8	3618	3608.9	3598.9	3587.8	3575.4	3561.7	3546.8
3rd	4199.6	4199.6	4199.6	4199.5	4199.5	4199.5	4199.4	4199.4	4199.4
4th	5933	5917.3	5900.3	5883	5864.6	5844.5	5820.8	5344	4852.9

**Table 3:** Maximum stress in six shock directions of shock response spectrum.

The Thickness of V-Shaped Beam (μm)	The Maximum Stress Value Generated by Six Shock Directions (MPa)					
	X+ Direction	X– Direction	Y+ Direction	Y− Direction	Z+ Direction	Z− Direction
100	43.0857	39.2143	0.8280	0.8630	83.0453	81.5029
95	41.6550	39.1881	0.8264	0.7865	83.1088	84.3100
90	35.8839	39.5759	0.7280	0.7478	86.8901	86.1178
85	38.5236	38.4510	0.7376	0.7353	93.0485	93.0750
80	35.6123	37.9682	0.6950	0.7007	94.1470	94.7207
75	40.8235	39.2736	0.6337	0.6234	102.0094	100.5120
70	53.9320	50.8894	0.5912	0.5910	114.2333	114.2545
65	8.1650	8.1667	0.5824	0.5684	129.8858	127.5013
60	7.8315	7.8165	0.5201	0.5472	142.1292	149.0320

**Table 4:** Comparison of existing shock resistance technology and this gyroscope.

	Type	Shock Resistance
BEI Systron Donner	QRS11	200 g, ½ sine pulse
	QRS28	300 g, 5 ms, ½ sine pulse
	QRS2000	400 g, 2 ms, ½ sine pulse
	QRS14	200 g, ½ sine pulse
	SDG1400	200 g, 2 ms, ½ sine pulse
	QRS116	1000 g, 2 ms, ½ sine pulse
Watson Industries	ADS–C132	500 g, 10 ms, ½ sine pulse
	ADS–C232	
	ARS–G152	
Our Gyroscope	–	1500 g, 2 ms, ½ sine pulse
